# Glucose and lactate metabolism in the awake and stimulated rat: a ^13^C-NMR study

**DOI:** 10.3389/fnene.2013.00005

**Published:** 2013-05-31

**Authors:** Denys Sampol, Eugène Ostrofet, Marie-Lise Jobin, Gérard Raffard, Stéphane Sanchez, Véronique Bouchaud, Jean-Michel Franconi, Gilles Bonvento, Anne-Karine Bouzier-Sore

**Affiliations:** ^1^Centre de Résonance Magnétique des Systèmes Biologiques, CNRS-Université Bordeaux Segalen UMR 5536Bordeaux, France; ^2^Commissariat à l'Energie Atomique et aux Energies Alternatives, Département des Sciences du Vivant, Institut d'Imagerie Biomédicale, Molecular Imaging Research Center and CNRS CEA URA 2210Fontenay-aux-Roses, France

**Keywords:** ^13^C NMR spectroscopy, lactate, brain metabolism, neurons, astrocytes

## Abstract

Glucose is the major energetic substrate for the brain but evidence has accumulated during the last 20 years that lactate produced by astrocytes could be an additional substrate for neurons. However, little information exists about this lactate shuttle *in vivo* in activated and awake animals. We designed an experiment in which the cortical barrel field (S1BF) was unilaterally activated during infusion of both glucose and lactate (alternatively labeled with ^13^C) in rats. At the end of stimulation (1 h) both S1BF areas were removed and analyzed by HR-MAS NMR spectroscopy to compare glucose and lactate metabolism in the activated area vs. the non-activated one. In combination with microwave irradiation HR-MAS spectroscopy is a powerful technical approach to study brain lactate metabolism *in vivo*. Using *in vivo* ^14^C-2-deoxyglucose and autoradiography we confirmed that whisker stimulation was effective since we observed a 40% increase in glucose uptake in the activated S1BF area compared to the ipsilateral one. We first determined that lactate observed on spectra of biopsies did not arise from post-mortem metabolism. ^1^H-NMR data indicated that during brain activation there was an average 2.4-fold increase in lactate content in the activated area. When [1-^13^C]glucose + lactate were infused ^13^C-NMR data showed an increase in ^13^C-labeled lactate during brain activation as well as an increase in lactate C3-specific enrichment. This result demonstrates that the increase in lactate observed on ^1^H-NMR spectra originates from newly synthesized lactate from the labeled precursor ([1-^13^C]glucose). It also shows that this additional lactate does not arise from an increase in blood lactate uptake since it would otherwise be unlabeled. These results are in favor of intracerebral lactate production during brain activation *in vivo* which could be a supplementary fuel for neurons.

## Introduction

Glucose is the main blood substrate to provide energy to the central nervous system. However, alternative substrates other than glucose can be used by the brain. As early as 1991, two groups observed that during photic stimulation, a lactate peak was transiently observed in the human visual cortex using localized ^1^H-NMR spectroscopy, and that it disappeared in the dark (Prichard et al., 1991; Sappey-Marinier et al., 1992). Both concluded that this phenomenon could be due to a transient increase in glycolysis over respiration during stimulation. However, a more detailed explanation was given in 1994 with the observation that when primary astrocytes in culture are exposed to glutamate, they increase their glucose consumption and produce a significant amount of lactate that is released in the medium (Pellerin and Magistretti, 1994). An astrocyte-neuron lactate shuttle (ANLS) was proposed, a concept that describes a coupling mechanism between neuronal activity and glucose utilization and involving activation of aerobic glycolysis in astrocytes and lactate consumption by neurons. Several articles collecting experimental evidence as well as theoretical arguments in favor or against this model have been written (Bouzier-Sore et al., 2002; Chih and Roberts, 2003; Hertz, 2004; Bonvento et al., 2005; Pellerin et al., 2007; Dienel, 2012; Pellerin and Magistretti, 2012). More recently, another molecular pathway involving soluble adenylyl cyclase was proposed to play a role in this metabolic coupling between neurons and astrocytes (Choi et al., 2012). Whatever the mechanism to couple astrocyte and neuron metabolism, brain lactate has become an issue for debate among scientists in the last 15 years (Pellerin et al., 1998; Schurr and Rigor, 1998; Magistretti and Pellerin, 1999; Chih et al., 2001; Dienel and Hertz, 2001).

^13^C-NMR spectroscopy is a powerful method to analyze brain metabolism and to study metabolic interactions between neurons and astrocytes. The first studies using NMR spectroscopy were performed *in vitro*. Neurons were incubated with various ^13^C-labeled substrates. When incubated with [1-^13^C]glucose, the latter was shown to be an efficient substrate for the neurons (Cruz et al., 2001) and surprisingly to a lesser extent than astrocytes in another study (Leo et al., 1993). When neurons were incubated with [3-^13^C]lactate, the latter was also shown to be an efficient substrate (Schousboe et al., 1997; Waagepetersen et al., 1998a,b). ^13^C-pyruvate (Brand et al., 1992; Cruz et al., 2001), ^13^C-aspartate (Bakken et al., 1998), and ^13^C-alanine (Zwingmann et al., 2000) have also proved to be neuronal substrates. For each experiment, the ^13^C of the labeled substrate was incorporated into different metabolites and amino acids, indicating that the labeled substrate was metabolized. Therefore, no clear difference was evidenced at that time between glucose and lactate metabolism in neuronal cultures. It seems that whatever the substrate present in the medium, the cell has no choice other than to adapt and to metabolize it, a well-known phenomenon. In response to these studies, a competition experiment between glucose and lactate was performed (Bouzier-Sore et al., 2003, 2006). Both glucose and lactate, which were alternatively ^13^C-labeled, were added to the culture neuronal medium, a much more physiological condition since both molecules are present in brain tissue. The results clearly indicated that lactate was preferentially used for the neuronal oxidative metabolism compared to glucose. Moreover, when injected intravenously to anesthetized rats, [3-^13^C]lactate was found to be metabolized only in neurons (Bouzier et al., 2000; Hassel and Brathe, 2000). However, *in vitro* experiments do not reflect what occurs *in vivo*, and the latter-mentioned *in vivo* studies were performed on anesthetized animals, a situation not entirely reflecting the metabolism occurring during brain activation.

We designed an experiment where ^13^C-labeled substrate was infused in awake rats in which unilateral whisker stimulation was performed. This stimulation leads to activation of a local brain area called the barrel field or S1BF region, which is located in the somatosensory cortex. This protocol has two major advantages. First, the S1BF is a brain area in which the neuronal anatomy and circuitry are widely documented (Giaume et al., 2009). Neurons are arranged in clusters called barrels. Each barrel is an element of a functional column that spreads from layers II and III to layer IV and receives inputs from its corresponding whisker on the contralateral side of the rat face: one right whisker activates one left barrel. Since we performed unilateral stimulation, the second advantage was to obtain an internal control for each rat, i.e., the right S1BF at rest.

Using ^14^C-2-deoxyglucose, we first checked that whisker stimulation could be observed in the corresponding S1BF area. Then, awake rats were infused with either a [1-^13^C]glucose + lactate, or a glucose + [3-^13^C]lactate solution during stimulation. After a rapid euthanasia (less than 1 s) using microwaves focused on the brain, both left (activated) and right (rest) S1BF areas were dissected and further analyzed by ^1^H- and ^13^C-NMR spectroscopy.

## Materials and methods

### Animals and infusion techniques

The experimental protocols used in this study were approved by appropriate institutional review committees, met the guidelines of the appropriate governmental agency and were performed by persons having their own animal experiment accreditation (authorization n°33 10 003).

#### Infusion protocol

Female Wistar rats (200 ± 10 g) were used in all experiments (*n* = 14). Animals were slightly held on a Plexiglas support during stimulation. Therefore, before infusion, each animal was accustomed to the experimental set-up (at least 3 times, 1 h) to reduce the stress of contention. Once rats were lying quietly, they were prepared for infusion. Right whiskers were mechanically stimulated at a rate of 5 Hz for 1 h. Infusions were performed in the tail vein for 1 h during whisker stimulation to reach the isotopic steady state. Rats were infused with a solution containing either [1-^13^C]glucose (750 mM, Cambridge isotope, 99% enrichment) + lactate (534 mM) or glucose (750 mM) + [3-^13^C]lactate (534 mM, Cambridge isotope, 98% enrichment). Intravenous infusions were carried out using a syringe pump that allows a flux such as glucose and lactate concentrations in the blood to remain constant. The infusate flow was monitored to obtain a time-decreasing exponential from 15 to 1.23 mL/h during the first 25 min, after which the rate was kept unchanged. At the end of the experiment, a sample of blood was removed. Rats were rapidly euthanized by cerebral-focused microwaves (5 KW, 1 s, Sacron8000, Sairem), the only way to immediately stop all enzymatic activities. This method avoids post-mortem artifacts such as anaerobic lactate production.

S1BF areas (right non-activated and left-activated) were dissected out using a rat brain matrix that allows precise and reproducible sampling of small brain regions, dipped in liquid nitrogen and kept at –80°C for further NMR analyses. Both activated and non-activated S1BF areas were analyzed by HR-MAS NMR spectroscopy on a Bruker Advance 500 MHz, directly on biopsies or after perchloric acid extracts. HR-MAS, high resolution at magic angle spinning, allowed spectra to be obtained directly on biopsies (50 μ g) or on small perchloric acid extracts (50 μ l). Moreover, the use of cerebral-focused microwaves is a method of choice since no post-mortem metabolism occurs (spectra were compared and lactate was quantified after either microwave, funnel freezing or decapitation under sodium pentobarbital overdose euthanasia).

#### Blood kinetics

During the 60-min infusion, blood samples (200–300 μ L, femoral artery) were periodically collected (at *t* = 0, 5, 10, 20, 30, and 60 min) to determine glucose and lactate concentrations as well as glucose- and lactate-specific ^13^C-enrichments. Experiments were performed both on awake (*n* = 4) and anesthetized animals (intraperitoneal injection of chloral hydrate, 8%, 0.5 ml/100 g of body weight, *n* = 4).

### Measurement of cerebral glucose uptake

Cerebral glucose uptake was measured as previously described (Cholet et al., 1997) using the (^14^C)2-deoxyglucose autoradiographic technique (Sokoloff, 1981) during mechanical stimulation (5 Hz) of the right whiskers. Rats (*n* = 2) were habituated to rest quietly on a Plexiglas support. Tygon catheters were inserted into the femoral vein and artery under isoflurane anesthesia. One hour after recovery from anesthesia, rats were injected i.v. with a (^14^C)2-deoxyglucose solution (125 μ Ci/100 g in 0.9 ml NaCL 0.9%) 5 min after initiation of whisker stimulation. Body temperature was monitored and maintained at 37.5°C during the experiment. At the end of the 45-min period, the animal was euthanized by a rapid blood injection of sodium pentobarbital. The brain was immediately removed and frozen, and 20 μm-thick coronal sections were processed for autoradiography using ^14^C standards (ARC 146C).

### Image analysis

Analysis of autoradiograms was performed using a computer-based image analysis system (MCID). The metabolic response to whisker stimulation was calculated as the ratio of the 2DG uptake measured in activated and resting S1BF.

### Brain perchloric acid extracts

The frozen cerebral tissues were weighed (around 30 mg) and a volume of 1 mL of 0.9 M perchloric acid was added. Sonication was then performed (4°C), followed by centrifugation (5000 g, 15 min, 4°C). The supernatant was neutralized with KOH to pH = 7.2 and centrifuged once more to eliminate potassium perchlorate salts. Samples were lyophilized and dissolved in 100 μL D_2_O containing ethylene glycol (0.04 M, peak at 63 ppm).

### NMR spectroscopy

#### ^1^H-NMR spectroscopy

NMR spectra were obtained with a Bruker DPX500 spectrometer equipped with an HRMAS probe. ^1^H-NMR spectra were acquired at 4°C with 90° flip angle (measured for each sample), 8 s relaxation delay, 5000 Hz sweep width and 32 K memory size. Residual water signal was suppressed by homonuclear presaturation. The ^13^C-enrichment of lactate C3 and that of glucose C1 in rat sera and tissue extracts were determined from satellite peak areas resulting from the heteronuclear spin-coupling patterns on spectra.

#### ^13^C-NMR spectroscopy

Proton-decoupled ^13^C-NMR spectra were acquired overnight using 60° flip angle, 20 s relaxation delay, 25063 Hz sweep width and 64 K memory size. Measurements were conducted at 4°C under bi-level broadband gated proton decoupling and D_2_O lock. From these spectra, the ^13^C content at the different carbon positions of compounds was determined as previously described (Bouzier et al., 1999). Briefly, incorporation of ^13^C into the different carbons in glutamate, glutamine, GABA, alanine, aspartate and lactate were determined from integration of the observed resonances relative to the ethylene glycol peak (63 ppm, external reference, known amount). Perchloric acid extract spectra were normalized thanks to ethylene glycol and protein contents.

#### Proton-observed carbon-editing (POCE) sequence

A POCE sequence was used to determine the ^13^C-specific enrichment at selected metabolite carbon positions using the (^13^C-^1^H) heteronuclear multiquanta correlation (Freeman et al., 1981; Rothman et al., 1985). The sequence enabled the successive acquisitions of a first scan corresponding to a standard spin-echo experiment without any ^13^C excitation and a second scan involving a ^13^C inversion pulse allowing coherence transfer between coupled ^13^C and ^1^H nuclei. Subtraction of two alternate scans resulted in the editing of ^1^H spins coupled to ^13^C spins with a scalar coupling constant *J*_CH_ = 127 Hz. ^13^C decoupling during the acquisition collapsed the ^1^H−^13^C coupling to a single ^1^H resonance. Flip angles for rectangular pulses were carefully calibrated on both radiofrequency channels before each experiment. The relaxation delay was 8 s for a complete longitudinal relaxation. The fractional ^13^C-enrichment was calculated as the ratio of the area of a given resonance on the edited ^13^C-^1^H spectrum to its area on the standard spin-echo spectrum. The reproducibility and accuracy of the method were assessed using several mixtures of ^13^C-labeled amino acids and lactate with known fractional enrichments. Both were better than 5%.

### Determination of proteins, glucose and lactate contents

Protein content was determined according to the procedure of Lowry et al. (1951) using bovine serum albumin as standard.

Blood glucose concentrations were determined by enzymatic assays using the coupled reactions of glucose oxidase and peroxidase. Blood lactate concentrations were determined using lactate dehydrogenase (Sigma).

### Statistical analysis

Data are given as mean ± SD values. They were analyzed by Student's *t*-test or the paired *t*-test. The level of significance was set at *p* < 0.05.

## Results

### Blood lactate and glucose evolutions during infusion

**Figure 1:**
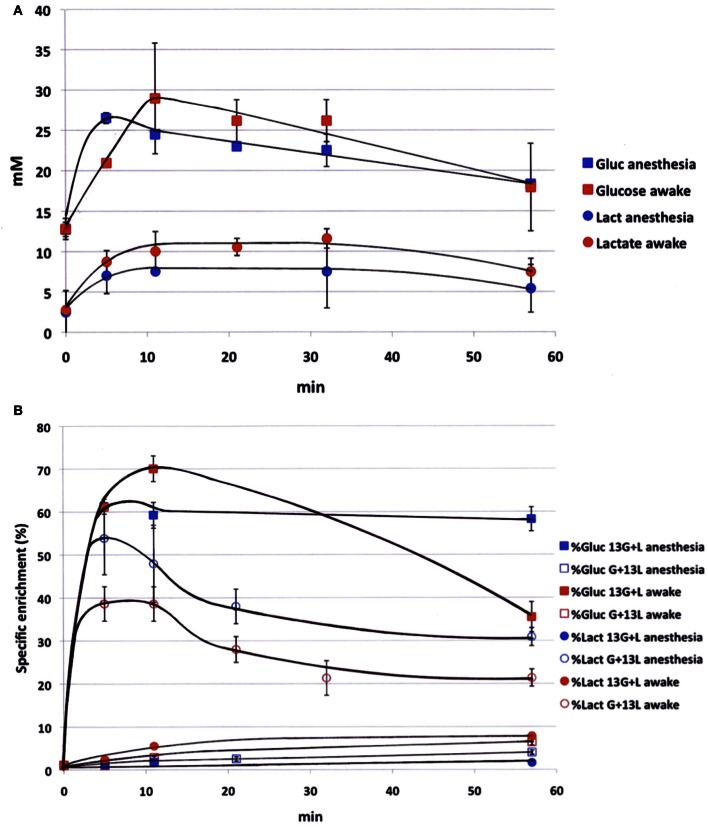
**Time evolution of labeled glucose and lactate during infusion. (A)** Glucose (squares) and lactate (circles) concentrations (mM) were measured during infusion (glucose 750 mM and lactate 534 mM), both in anesthetized (blue) and awake (red) rats; *n* = 4. No statistical difference (except for glucose concentration, time point 5 min). **(B)** [1-^13^C]glucose (squares) and [3-^13^C]lactate (circles) specific enrichments (%) were measured during infusion with [1-^13^C]glucose + lactate (filled dots, *n* = 7) or glucose + [3-^13^C]lactate (open dots, *n* = 7), both in anesthetized (blue) and awake (red) rats. *p* < 0.05 between awake and anesthetized rats at the end of the infusion.

Blood samples were collected periodically during infusion to determine the time evolution of both glucose and lactate concentrations (Figure 1A) and variations in ^13^C-enrichments of lactate C3 and glucose C1 (Figure 1B). Experiments were conducted both on anesthetized and awake rats in order to compare the kinetics of substrates in both conditions. Blood concentrations of glucose and lactate seemed to be slightly higher in awake rats compared to anesthetized ones, but no statistical difference (except for glucose, time point 5 min) was found. During [1-^13^C]glucose + lactate infusion, the specific ^13^C-enrichment of glucose C1 was lower in the awake animals (Figure 1B). A very small part of the [1-^13^C]glucose was converted into [3-^13C^]lactate in anesthetized rats (maximum value: 1.8% after 10 min), whereas this conversion was 4-fold greater in awake rats. During glucose + [3-^13^C]lactate infusion, the specific ^13^C-enrichment of lactate C3 was also lower in awake rats (Figure 1B). The specific enrichment of glucose C1 increased very slowly with infusion time, and more significantly in awake rats. At the end of infusion, it was 3.9% in anesthetized rats and 6.4% in awake rats.

### Validation of whisker activation

**Figure 2:**
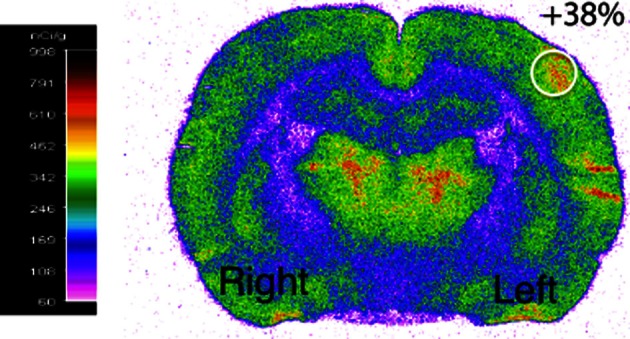
**Functional activation of the brain during whisker stimulation.** Digitized, pseudocolored autoradiogram of the distribution of ^14^C-2-deoxyglucose uptake on representative brain section from awake and unilaterally stimulated rat. The region of interest is represented by the white circle.

Functional activation was performed by stimulating the right whiskers (5 Hz, 45 min) in awake rats, which led to a significant increase (40 ± 2%) in cerebral glucose uptake in the left (activated) S1BF area compared to the right (rest) one (Figure 2). This result validated the stimulation protocol, which was then used during ^13^C-labeled substrate infusion in the next experiments. The same S1BF area were dissected and analyzed by NMR spectroscopy.

### Comparison of ^1^H-NMR spectra between different sacrifice procedures

**Figure 3:**
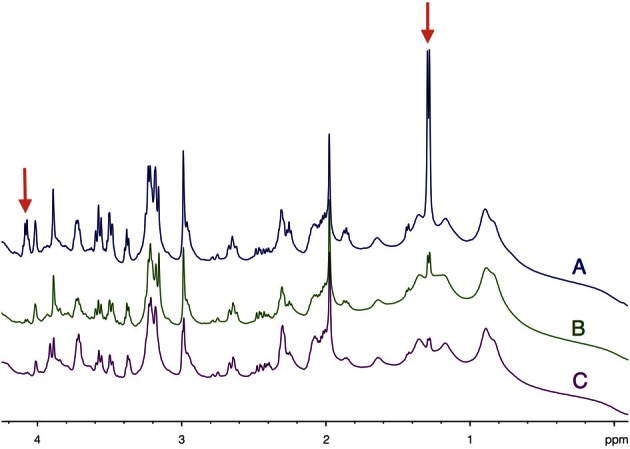
**Determination of brain tissue lactate content as function of the sacrifice procedure.** HRMAS ^1^H-NMR spectra of brain biopsies rapidly removed after euthanasia by **(A)** decapitation, **(B)** funnel freezing, and **(C)** focused microwaves. Lactate can be detected on the spectra (red arrows) at 1.32 ppm (doublet, protons linked to carbon 3) and at 4.11 ppm (quadruplet, proton linked to carbon 2). Spectra were normalized thanks to biopsie weights and fumarate peak (6.9 ppm), added as an external reference.

HRMAS ^1^H-NMR spectra of brain biopsies were compared with three methods of sacrifice: decapitation (Figure 3A), funnel freezing (Figure 3B) and rapid microwave fixation of the brain (Figure 3C). In all cases, brains were rapidly removed from the skull, dipped into liquid nitrogen and stored at −80°C before HRMAS analysis. Spectra were normalized to biopsy weights. Spectra clearly indicated that lactate peaks (red arrows) were the lowest after microwave fixation. There was a 19-fold increase in lactate content between the microwave and the decapitation biopsies, and a 2.2-fold increase between the microwave and the funnel freezing biopsies. When fumarate was added as an external reference, lactate concentration was estimated to be around 1 mM in the microwave biopsy. This method of euthanasia is therefore required to prevent post-mortem metabolism, and thus anaerobic lactate production.

### Comparison of ^1^H-NMR spectra between activated and rest areas

**Figure 4:**
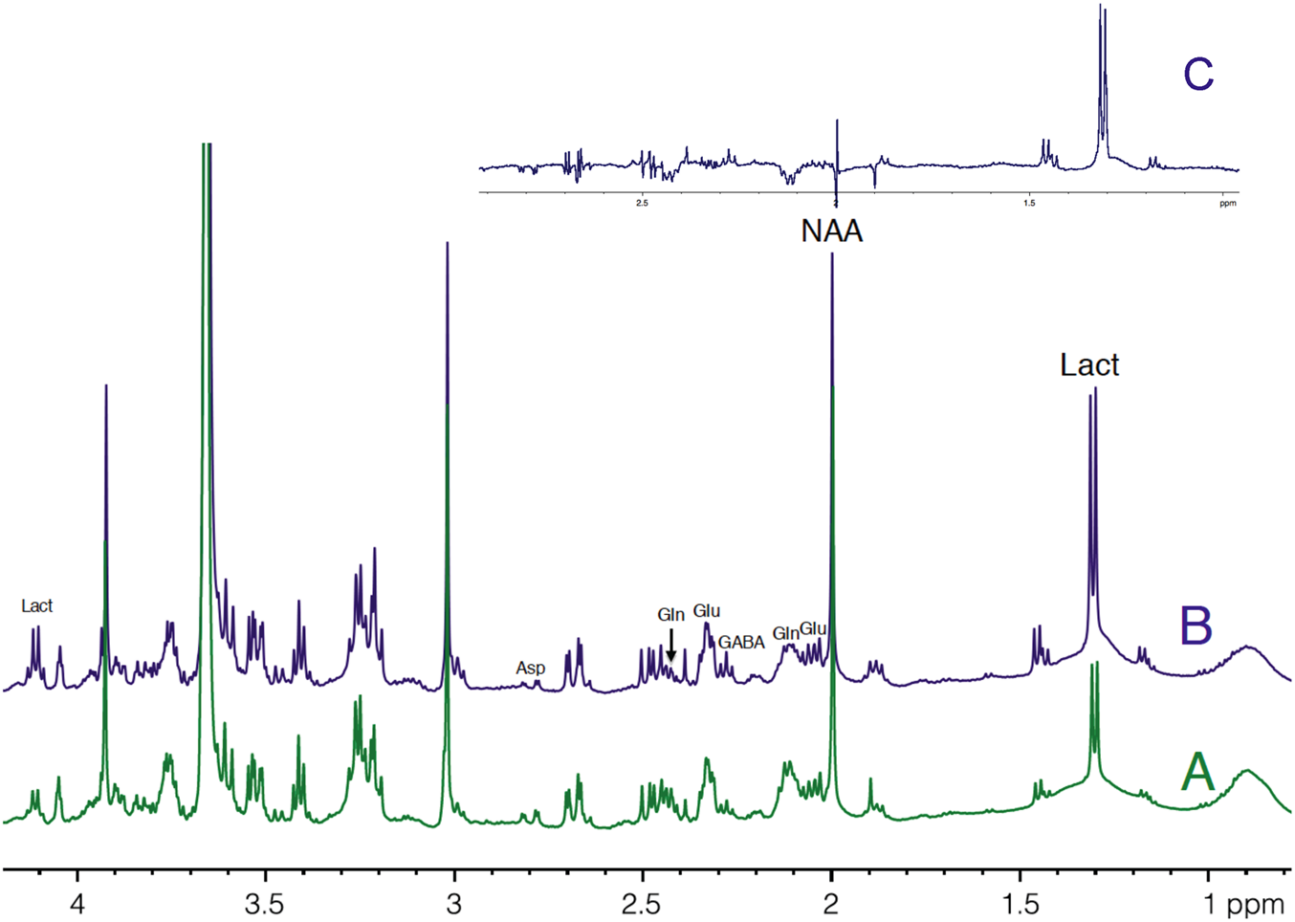
**HRMAS ^1^H-NMR spectra of perchloric acid extracts of rest (A) and activated (B) S1BF areas.** Spectra were obtained from rat n°5, in which ratio of lactate between rest **(A)** and activated **(B)** S1BF was 2.6, representing the closer the mean ratio in the 14 rats. The difference between the two spectra is presented in **(C)**. Ethylen glycol was added as an internal reference. Lact, lactate; NAA, N-acetyl-aspartate; Glu, glutamate; Gln, glutamine; Asp, aspartate.

HRMAS ^1^H-NMR spectra of perchloric acid extracts of S1BF biopsies from microwave-treated brains are presented in Figure 4. Spectra were normalized to protein content on the ethylene glycol peak, added as an external reference. Lactate content was determined and the ratio between activation and rest was measured in each rat (*n* = 14). In 12 out of 14 rats, lactate content in the activated S1BF area increased compared to the rest one, the lowest ratio being 1.4 and the highest being 4.1. In the two other rats, no difference was observed (ratios were 0.95 and 0.99). The mean value of the ratio of lactate contents (activated area over rest area) was 2.4.

### ^13^C incorporation into brain metabolites in activated and rest areas

The metabolism and fate of ^13^C from [1-^13^C]glucose and [3-^13^C]lactate is the same as both labeled substrates yields [3-^13^C]pyruvate (for a scheme of the labeling, see (Bouzier et al., 1999; Bouzier-Sore et al., 2002, 2006)). However, since one mole of [1-^13^C]glucose gives one mole of labeled [3-^13^C]pyruvate and one mole of unlabeled pyruvate, there is an isotopic dilution of 50% when starting from [1-^13^C]glucose, which is not the case when [3-^13^C]lactate is the labeled substrate (one mole of [3-^13^C]lactate gives one mole of [3-^13^C]pyruvate). From [3-^13^C]pyruvate, [3-^13^C]alanine can be generated. [3-^13^C]pyruvate enters the TCA cycle through two pathways: via the pyruvate dehydrogenase (PDH) or the pyruvate carboxylase (PC) route. According to the PDH pathway, [3-^13^C]pyruvate is converted to [2-^13^C]acetyl-CoA, which enters the TCA cycle to give [4-^13^C]citrate. Within the first TCA cycle turn, the label is further transferred to α-[4-^13^C]ketoglutarate and then to [2-^13^C]- or [3-^13^C]oxaloacetate. From α-[4-^13^C]ketoglutarate, [4-^13^C]glutamate and thereafter [4-^13^C]glutamine and [2-^13^C]GABA can be obtained, whereas [2-^13^C]- or [3-^13^C]oxaloacetate may give rise to [2-^13^C]- or [3-^13^C]aspartate. According to the PC pathway, which is present only in astrocytes (Norenberg and Martinez-Hernandez, 1979), [3-^13^C]pyruvate is converted into [3-^13^C]oxaloacetate, which enters the TCA cycle. Depending on the yield of cycling between oxaloacetate and fumarate, a fraction of the label can, however, be recovered as [2-^13^C]oxaloacetate (Merle et al., 1996). At the next TCA cycle turn, the label entered by the PC route is mainly recovered as α-[2-^13^C]ketoglutarate. From [3-^13^C]oxaloacetate and α-[2-^13^C]ketoglutarate, [3-^13^C]aspartate, [2-^13^C]glutamate and thereafter [2-^13^C]glutamine are then principally formed.

**Figure 5:**
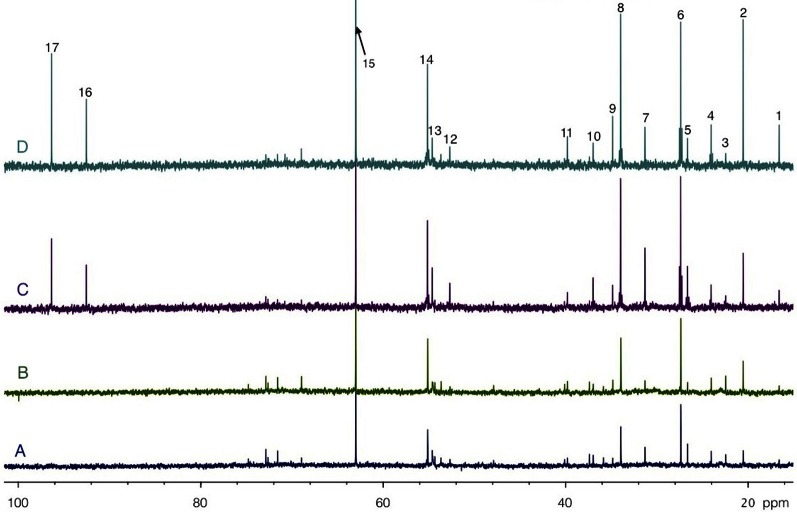
**HRMAS ^13^C-NMR spectra of perchloric acid extracts of rest (A and C) and activated (B and D) S1BF areas, after infusion with glucose + [3-^13^C]lactate (A and B) or [1-^13^C]glucose + lactate (C and D).** Spectra were normalized owing to their protein contents and to the EG peak (15, 63 ppm). Peak assignments: 1: alanine C3, 2: lactate C3, 3: NAA C6, 4: GABA C3, 5: glutamine C3, 6: glutamate C3, 7: glutamine C4, 8: glutamate C4, 9: GABA C2, 10: aspartate C3, 11: GABA C4, 12: aspartate C2, 13: glutamine C2, 14: glutamate C2, 15: EG, 16: glucose C1α, 17: glucose C1β.

Both [1-^13^C]glucose + lactate and glucose + [3-^13^C]lactate infusions induced the labeling of brain amino acids and lactate, as illustrated in the ^13^C-NMR spectra shown in Figure 5. These spectra were acquired on perchloric acid extracts of rest areas (Figures 5A,C) or activated areas (Figures 5B,D) after either a 1 h-infusion with glucose + [3-^13^C]lactate (Figures 5A,B) or with [1-^13^C]glucose + lactate (Figures 5C,D). Relative peak intensities and coupling figures were lower when glucose + [3-^13^C]lactate were infused than in the [1-^13^C]glucose + lactate condition, indicating a lower ^13^C incorporation. The lactate C3 peak was much higher in the activated area compared to the rest one in the [1-^13^C]glucose + lactate condition, whereas this increase was much lower in the glucose + [3-^13^C]lactate condition.

The specific enrichments of selected amino acid carbons were determined from spectra acquired with the POCE sequence. The values are reported in Table 1. Specific enrichment values were much higher in the [1-^13^C]glucose + lactate condition compared to the one measured after glucose + [3-^13^C]lactate infusion. In the latter condition, specific enrichments were indeed very low (natural abundance, 1.1%, was not subtracted). However, we found a statistical decrease in lactate C3-specific enrichment between rest and activated areas. After [1-^13^C]glucose + lactate infusion, lactate and alanine C3-specific enrichments were higher in the activated area compared to the rest one, whereas the opposite was found for glutamine C4.

**Table 1:** **Specific enrichments (%) of some carbon positions of brain metabolites, after infusion of [1-^13^C]glucose + lactate (^13^G + L) or glucose + [3-^13^C]lactate (G + ^13^L**).

**Specific enrichments (%)**			
	**^13^G + L**		**G + ^13^L**	
	**Rest**	**Activated**	**Rest**	**Activated**
Lact C3	10.2 ± 0.4	13.5 ± 2.9^*^	3.2 ± 0.8	2.7 ± 0.8^*^
Glu C4	19.1 ± 1.2	18.5 ± 2.4	4.0 ± 1.0	4.1 ± 0.9
Asp C3	15.5 ± 1.3	15.4 ± 3.0	2.6 ± 0.3	3.0 ± 1.3
GABA C2	18.3 ± 0.5	17.7 ± 1.1	2.6 ± 0.4	2.9 ± 0.3
Gln C4	14.2 ± 1.1	11.4 ± 2.6^*^	3.5 ± 0.9	3.6 ± 1.0
Ala C3	15.5 ± 1.5	16.9 ± 0.6^*^	3.8 ± 1.1	3.4 ± 0.9

* p < 0.03 between rest and activation, paired t-test.

The enrichments of the different carbons within glutamate and glutamine were evaluated relatively to carbon 4, the most labeled carbon (Table 2). In the [1-^13^C]glucose + lactate condition, no difference was found between glutamate C2 and C3 both at rest and during activation, whereas glutamine C2 was more enriched than C3, reflecting the PC activity, which is present only in astrocytes. In the glucose + [3-^13^C]lactate condition, no further difference between glutamine C2 and C3 could be evidenced, as shown previously (Bouzier et al., 2000), and indicating that [3-^13^C]lactate is more a neuronal substrate (no PC activity).

**Table 2:** **Relative enrichments of glutamate and glutamine, after infusion of [1-^13^C]glucose + lactate (^13^G + L) or glucose + [3-^13^C]lactate (G + ^13^L**).

	**Relative enrichments**			
	**Glu**		**Gln**	
	**Rest**	**Activated**	**Rest**	**Activated**
**^13^G + L**				
C4	1	1	1	1
C2	0.72 ± 0.04	0.76 ± 0.04	0.81 ± 0.07	0.86 ± 0.07
C3	0.75 ± 0.02	0.76 ± 0.05	0.63 ± 0.04^#^	0.70 ± 0.06^#^
**C2/C3**	**0.96 ± 0.03**	**0.99 ± 0.05**	**1.28 ± 0.07°**	**1.24 ± 0.07°**
**G + ^13^L**				
C4	1	1	1	1
C2	0.95 ± 0.08	0.87 ± 0.10	0.91 ± 0.16	0.65 ± 0.20
C3	1.02 ± 0.16	0.92 ± 0.12	0.95 ± 0.08	0.68 ± 0.05
**C2/C3**	**0.93 ± 0.12**	**0.95 ± 0.11**	0.96 ± 0.12^*^	0.95 ± 0.13^*^

Enrichments of carbon 2 and 3 were measured relative to the most enriched carbon, which is carbon 4.

# p < 0.05 between C3 and C2; °p < 0.05 between Glu and Gln C2/C3 ratios;

* p < 0.05 between ^13^G + L and G + ^13^L infusions.

The ratio C2/C3 (in bold) was then calculated and reflects the contribution of the pyruvate carboxylase (when PC activity is present, C2/C3 > 1).

### Correlation between lactate concentration and lactate C3-specific enrichment increases during brain activation

**Figure 6:**
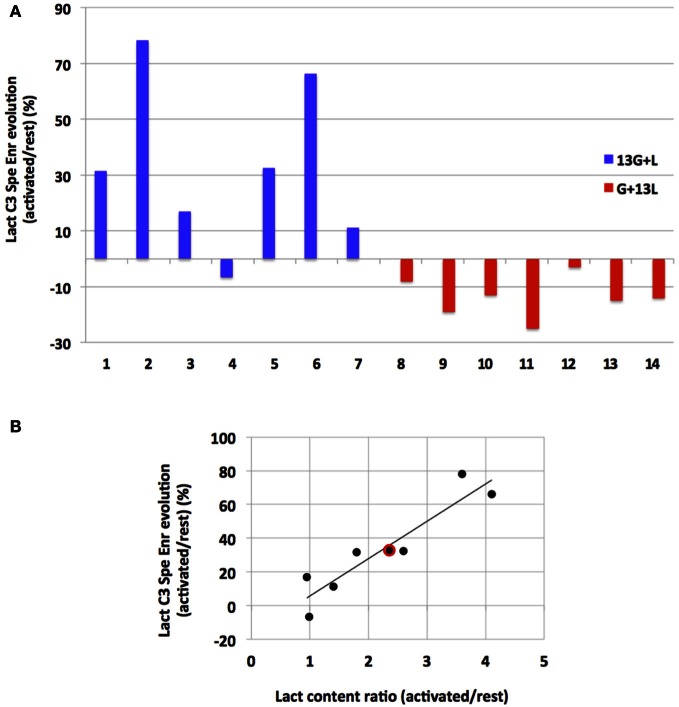
**Evolution of lactate C3 specific enrichment for each rat (A) and correlation between its evolution and lactate content during activation (B). (A)** Evolutions of lactate C3 specific enrichment (activated over rest, %) are presented for each independent experiment after infusion with [1-^13^C]glucose+lactate (*n*=7, rat 1 to rat 7, blue) or with glucose + [3-^13^C]lactate (*n*=7, rat 8–14, red). **(B)** Linear regression between lactate concentration increase (ratio between lactate content measured in the activated S1BF and the one measured in the rest S1BF on ^1^H-NMR spectra) and lactate C3 specific enrichment increases (values in **A**, blue) for the [1-^13^C]glucose + lactate condition (rat 1–7). Red dot represents the mean values in this latter condition (a 2.4-fold increase in the lactate content, and a 33% increase in lactate C3 specific enrichments). *r*^2^= 0.86.

To our knowledge, this study is the first in which ^13^C-labeled substrate infusion was performed in awake and stimulated rats. This protocol was used to mimic physiological states as closely as possible. However, since the rats were awake, they were able to move their heads freely, leading to inter-individual variations. It was thus interesting to compare results obtained for each independent experiment (Figure 6). When infused with [1-^13^C]glucose + lactate, 6 out of 7 rats demonstrated an increase in lactate C3-specific enrichment. When infused with glucose + [3-^13^C]lactate, all rats underwent a decrease in lactate C3-specific enrichment, even if the enrichment values were low.

Interestingly, we found a correlation (*r*^2^= 0.86) between the increase in lactate concentration during activation (measured on ^1^H-NMR spectra and reflecting the total lactate content in the barrel field biopsy, at rest or activated) and the increase in lactate C3-specific enrichment measured between rest and activated states (when [1-^13^C]glucose + lactate was infused).

## Discussion

The time course of glucose and lactate concentrations and that of glucose C1 and lactate C3 ^13^C-enrichments (Figure 1) emphasize the intricate glucose-lactate metabolic relationship already shown in a previous study (Bouzier et al., 2000) and demonstrated here to be even more complex when rats are awake. Results indicated an active blood lactate metabolism, as already reported (Jenkins et al., 1993), and a recycling attributed to liver gluconeogenesis, which was greater in awake than in anesthetized rats. However, this conversion is minimized by the addition of glucose in the [3-^13^C]lactate infusion solution (Bouzier et al., 2000). In parallel, blood glucose can also be converted into lactate, as confirmed by the higher lactate concentration in awake rats than in anesthetized ones, as well as by the specific evolution in enrichment of [3-^13^C]lactate, i.e., an increase after [1-^13^C]glucose + lactate infusion and a decrease in the glucose + [3-^13^C]lactate condition. Therefore, working with awake and stimulated animals modifies glucose and lactate blood kinetics. However, in both infusion conditions, we were able to measure specific enrichment values and to find statistical differences between rest and stimulated S1BF in awake animals.

^1^H-NMR spectra showed a mean 2.4-fold increase in lactate content during brain activation. This lactate increase has already been observed in human visual cortex during photic stimulation using localized ^1^H-NMR spectroscopy (Prichard et al., 1991; Sappey-Marinier et al., 1992; Mangia et al., 2007) as well as in rats using microdialysis (Caesar et al., 2008). In the present study, the brain irradiation using focused microwaves was a key element. Indeed, Figure 3 clearly shows that the lactate detected on our ^1^H-NMR spectra was the physiologic lactate present in brain tissue and was not that arising from post-mortem or anaerobic glycolysis. Note that the funnel freezing technique, the classical euthanasia procedure when studying brain metabolism, induced a 2.2-fold increase in lactate content compared to microwave irradiation. The lactate concentration in brain tissue relative to the NAA peak was found to be around 1 mM in the absence of activation, a value in accordance with data obtained either by microdialysis or NMR spectroscopy (Prichard et al., 1991; Fellows et al., 1992; Abi-Saab et al., 2002).

^13^C-NMR spectra (Figure 5) indicate that the ^13^C-labeled substrate entered the brain and was metabolized in both infusion conditions. ^13^C was incorporated into amino acids such as glutamine, glutamate, alanine, aspartate and GABA. However, this incorporation was much lower when [3-^13^C]lactate was the labeled substrate, leading to low specific enrichment values. Even under such conditions, variations between rest and activated states were statistically observed for lactate C3-specific enrichments. When resting and activated states were compared for each independent experiment (Figure 6), an increase in lactate C3-specific enrichment was observed in 6 out of 7 rats infused with [1-^13^C]glucose + lactate, as well as a decrease in all rats infused with glucose + [3-^13^C]lactate. This result indicates that during brain activation, glucose uptake increases (as confirmed by the 2-DG experiment), as well as endogenous lactate synthesis. When glucose is ^13^C-labeled, lactate C3-specific enrichment also increases. When unlabeled glucose + [3-^13^C]lactate is infused, the same occurs: glucose uptake and lactate production increase. However, since the glucose is unlabeled, unlabeled lactate is synthesized and leads to the isotopic dilution of the [3-^13^C]lactate also present in the infusion solution.

This study was performed in awake rats, so inter-individual variations were to be expected. Even though barrel fields can be activated in anesthetized animals (Castro-Alamancos, 2004; Krupa et al., 2004; Chakrabarti and Alloway, 2009), we wanted to avoid any modification in brain metabolism due to anesthetics. This inter-individual variation could be accounted for by more or less efficient whisker activation. Indeed, rats were awake and able to freely move their heads, even if restrained. The quieter the rat, the more whiskers were stimulated. Interestingly, a correlation between lactate concentration increase (measured on ^1^H-NMR spectra and reflecting the total lactate content in the biopsy containing the barrel field) and changes in lactate C3-specific enrichments between rest and activated states (when [1-^13^C]glucose + lactate was infused) was obtained, confirming that the lactate appearing in the barrel field during brain activation originated from [1-^13^C]glucose breakdown (*r*^2^= 0.86) in an activity-dependent manner, as already proposed (Wyss et al., 2011).

When glucose + [3-^13^C]lactate were the substrates, we also observed a higher lactate C3 peak on the ^13^C-NMR spectrum in the activated S1BF, even if this increase was much lower than in the [1-^13^C]glucose + lactate condition. This was due to a combination of an increase in total lactate content and a decrease in lactate C3-specific enrichment, fewer lactate molecules being ^13^C-labeled. This confirms that lactate newly synthesized during brain activation arises from glucose (unlabeled in this case).

Like lactate C3, alanine C3-specific enrichment increased during brain activation under the condition [1-^13^C]glucose + lactate infusion, which is not surprising as both metabolites are at the same metabolic node linked to pyruvate. On the contrary, glutamine C4-specific enrichment decreased during activation. This indicates that during whisker stimulation, the ^13^C arising from glucose, which ends up in [3-^13^C]pyruvate at the end of the glycolysis, is less incorporated into the astrocytic TCA cycle and more probably converted into lactate by lactate dehydrogenase.

Concerning the relative enrichments, a difference in ^13^C-incorporation into glutamine carbon 2 and 3 is always detected after [1-^13^C]glucose infusion, thus reflecting the PC activity present only in astrocytes (Norenberg and Martinez-Hernandez, 1979). This astrocytic enzyme leads to a higher enrichment of carbon 2 compared to carbon 3 (Table 1, Gln, ^13^G + L) (Bouzier et al., 1999). This imbalance disappears (Table 1, Gln, ^13^G + L compared to G + ^13^L) when lactate is the ^13^C-labeled substrate. No further difference in glutamine carbon 2 and 3 was detected. The absence of a higher ^13^C incorporation in glutamine carbon 2 compared to carbon 3 when [3-^13^C]lactate is the labeled substrate reflects its metabolism in a PC-deprived compartment, i.e., in neurons. This specificity of lactate to be a neuronal substrate was already shown after [3-^13^C]lactate infusion in anesthetized rats (Bouzier et al., 2000) and in humans (Boumezbeur et al., 2010). In another study where [3-^13^C]lactate was administered in awake rats, the authors concluded that it enters the glutamatergic neurons preferentially (Qu et al., 2000).

The fact that lactate levels reliably increase with the onset of brain activation implies that regional glycolysis exceeds lactate utilization and/or clearance. Several *in vivo* studies using microdialysis (Caesar et al., 2008) or MRS (Mangia et al., 2007) have also shown an increase in lactate level that persists during the activation period, and not only during the first phase of brain activation. In our study, the whiskers were stimulated for 1 h, so it may be argued that a new higher steady state of lactate concentration is achieved during such sustained activation. This new steady state suggests that, in addition to being an energetic substrate, lactate could also have another role such as regulator of the cerebral blood flow (Mintun et al., 2004; Gordon et al., 2008). Indeed, in the latter studies, lactate was proposed to facilitate vasodilatation and increase cerebral blood flow. Recently, lactate was also suggested to act as a mediator of metabolic information for synapses (Bergersen and Gjedde, 2012).

The present findings clearly show that during brain activation, there is an increase in glucose uptake, an increase in glucose consumption and an increase in the production of lactate newly synthesized from blood glucose in the activated area. However, we were unable to determine in which compartment this metabolic conversion occurs; in astrocytes, as proposed by the ANLSH, or in neurons, as suggested by others in studies based on *in vitro* experiments (Bak et al., 2006). Nevertheless, in another study where the same whisker-to-barrel system was studied using a different approach (Voutsinos-Porche et al., 2003), the authors used 2DG autoradiography to establish that the enhanced glucose uptake during activation is dependent on the glutamate uptake occurring in astrocytes. More recently, Chuquet et al. (2010) using a fluorescent analog of 2DG and two-photon microscopy showed that the enhancement of glucose uptake in the barrel cortex upon whisker stimulation takes place essentially in the astrocytes. Combined with these previous findings, our results support the idea of a metabolic cooperation between the different cell types in the brain, i.e., astrocytes and neurons, the former becoming more glycolytic and the latter more oxidative during brain activation. Such metabolic interaction between neighboring cells is not unique as it has also been observed between different muscle fiber types (Brooks, 2009). Even if glucose is the main energetic substrate in adult mammals, lactate can no longer be considered as a metabolic end product but rather as a complementary and efficient substrate synthesized by one cellular type to supply energy to its neighbor.

### Conflict of interest statement

The authors declare that the research was conducted in the absence of any commercial or financial relationships that could be construed as a potential conflict of interest.

## Abbreviations

TCA: tricarboxylic acid
EG: ethylene glycol
NAA: N-acetyl-L-aspartate
POCE: proton-observed ^13^C-editing.
